# Circularly polarized light-sensitive, hot electron transistor with chiral plasmonic nanoparticles

**DOI:** 10.1038/s41467-022-32721-2

**Published:** 2022-08-29

**Authors:** Seok Daniel Namgung, Ryeong Myeong Kim, Yae-Chan Lim, Jong Woo Lee, Nam Heon Cho, Hyeohn Kim, Jin-Suk Huh, Hanju Rhee, Sanghee Nah, Min-Kyu Song, Jang-Yeon Kwon, Ki Tae Nam

**Affiliations:** 1 Department of Materials Science and Engineering, Seoul National University, Seoul, 08826 Republic of Korea; 2 Soft Foundry, Seoul National University, Seoul, 08826 Republic of Korea; 3 Seoul Center, Korea Basic Science Institute, Seoul, 02841 Republic of Korea; 4 School of Integrated Technology, Yonsei University, Incheon, 21983 Republic of Korea

**Keywords:** Nanoparticles, Nanoparticles, Nanophotonics and plasmonics, Nanophotonics and plasmonics, Electronic devices

## Abstract

The quantitative detection of circularly polarized light (CPL) is necessary in next-generation optical communication carrying high-density information and in phase-controlled displays exhibiting volumetric imaging. In the current technology, multiple pixels of different wavelengths and polarizers are required, inevitably resulting in high loss and low detection efficiency. Here, we demonstrate a highly efficient CPL-detecting transistor composed of chiral plasmonic nanoparticles with a high Khun’s dissymmetry (g-factor) of 0.2 and a high mobility conducting oxide of InGaZnO. The device successfully distinguished the circular polarization state and displayed an unprecedented photoresponsivity of over 1 A/W under visible CPL excitation. This observation is mainly attributed to the hot electron generation in chiral plasmonic nanoparticles and to the effective collection of hot electrons in the oxide semiconducting transistor. Such characteristics further contribute to opto-neuromorphic operation and the artificial nervous system based on the device successfully performs image classification work. We anticipate that our strategy will aid in the rational design and fabrication of a high-performance CPL detector and opto-neuromorphic operation with a chiral plasmonic structure depending on the wavelength and circular polarization state.

## Abstract (web summary)

One challenge with using circularly polarized light is an increased device complexity typically required to provide sufficient sensitivity to the polarization. Here, by integrating chiral plasmonic nanoparticles with a InGaZnO hot electron transistor, the authors present a polarization sensitive, optically active transistor.

## Introduction

Circularly polarized light (CPL) can be a good medium for deciphering high-order protein structures, understanding atmospheric conditions, encoding information, and inducing a Faraday rotation effect, resulting in versatile usability in various applications, such as circular dichroism (CD)^1^, LiDAR^2^, optical communication^3^, polarization-dependent holography^4^, and magnetic recording^5^. Under these uses, precise detection of two different circularly polarized states of CPL, left circularly polarized (LCP) and right circularly polarized (RCP) light, is very important. However, current technology indispensably requires multiple optical components, which significantly diminish the intensity of incident light, resulting in low efficiency in CPL detection. The conventional polarization detector requires color filters and polarizers on top of photodiodes. At least three polarizers of 0°, 45° and 90° for each red, green, and blue filter are required to obtain the full information of the polarization state of visible light^6^. In contrast, a single nanoparticle with a chiral plasmonic property can respond to the specific wavelength corresponding to the plasmonic resonance and generate different amounts of hot electrons in accordance with the polarization state. In this way, the minimum dimensions and pixel size can be significantly reduced because a single nanoparticle can act as a color filter and a polarizer simultaneously. In addition, a hot electron-based device can broaden the sensible spectral range because an energy lower than the optical bandgap can generate hot electrons at the Schottky junction^7,8^.

Photodetectors with two representative types have been demonstrated: photodiode and phototransistor. The diode type is widely adopted due to its simple device structure featuring two terminals; however, it only shows limited signal amplification. In contrast, the transistor type is a three-terminal device, in which additional gate voltage bias can control the carrier concentration of the semiconductor and the signal is amplified over a thousand times. Due to these controllability and amplification effects, the transistor has become the fundamental electronic component in various applications such as displays, memory, logic circuits, etc. To date, the hot electron-based photodetectors has been demonstrated as a diode, and as a result it has suffered low photoresponsivity. For example, the only literature addressing a hot electron-based CPL-detecting diode showed poor photoresponsivity in the range of a few mA/W (~4.3 mA/W (LCP), ~1.5 mA/W (RCP))^9^. To tackle this problem, the transistor device can be used as a breakthrough approach to achieve a high performance hot electron CPL detector. The key mechanism for hot electron photodetection depends on hot electron transport over a Schottky barrier, and only a transistor device can provide a new degree of freedom to finely control the barrier by manipulating the semiconductor conductance. Such effect can be quantitatively calculated as an activation energy according to gate voltage, and understanding the activation energy, especially depending on circular polarization states, can be a key parameter for achieving high performance a CPL detector using chiral plasmonic nanoparticles.

Beyond circular polarization sensing, hardware based opto-neuromorphic operation can be helpful for reducing computing power and time for imaging, classification and inference. Especially, the phototransistor device can be advantageous in the field of neuromorphic operation due to its synaptic plasticity modulation with light pulses^10,11^, and the artificial neural network (ANN) using phototransistor successfully emulates biological vision system featuring image recognition, contrast enhancement and noise reduction^12^. Such a synaptic characteristic is crucial for in-sensor computing and it significantly reduces latency and power consumption by eliminating analog to digital conversion and digital pre-processing stages^13–15^. In this system, controlling learning efficiency is important, and various degrees of freedom in the light source (wavelength, intensity, polarization, phase and, etc.), can possibly manipulate the learning efficiency of the synaptic device. In particular, the CPL detector using chiral plasmonic nanoparticles can demonstrate a differently learning synaptic device depending on circular polarization state; however, polarization dependent opto-neuromorphic function has not been explored yet.

Herein, we suggest a visible light operating CPL detector and its opto-neuromorphic operation with high photoresponsivity in which an InGaZnO (IGZO) transistor is used to effectively capture hot electrons from bottom-up processed plasmonic chiral gold nanoparticles. Under such a transistor structure, the chiral particles play the role of a wavelength- and circular polarization state-dependent filter, and IGZO is used as a hot electron acceptor for CPL detection. The CPL detector successfully distinguishes LCP light from RCP light in the visible light range, in which it shows high photoresponsivities of 1.37 A/W and 6.5 A/W (LCP) and 1.81 A/W and 3.62 A/W (RCP) at 635 nm and 780 nm excitation, respectively. Furthermore, the device shows neuromorphic characteristics based on CPL pulses and it shows different learning efficiency on image classification test. High-performance CPL detection is possible through hot electron generation of chiral gold nanoparticles and accelerated hot electron injection into the IGZO semiconductor by a gate voltage. The corresponding activation energy of the detector depending on the circular polarization state is analyzed, and the device shows activation energies of 890 meV and 610 meV (LCP) and 690 meV and 880 meV (RCP) under 635 nm and 780 nm excitation, respectively. More importantly, the CPL-dependent hot electron population is successfully investigated by femtosecond pump-probe spectroscopy, and the particles emit different numbers of hot electrons, by ~18–25%, depending on the circular polarization state under 650 nm pump laser excitation. We believe that this work not only advances the fundamental understanding of chiral plasmon-induced hot electron-based optoelectronics but also enables practical use of compact polarimeter and machine vision in integrated chips.

## Results and discussion

**Fig. 1:**
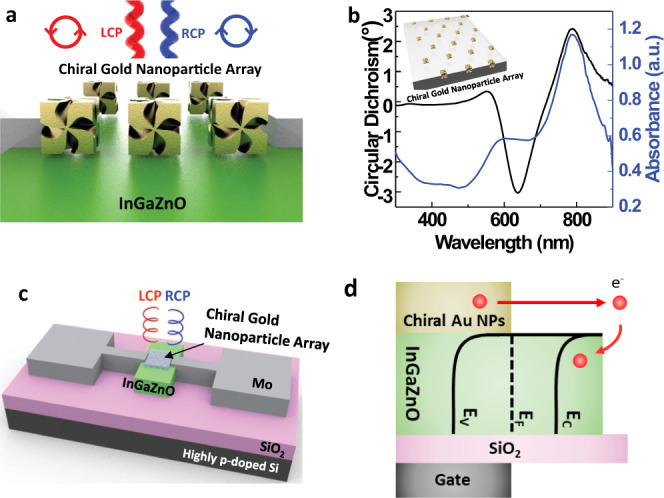
Schematic of the chiral gold nanoparticles and CPL-detecting transistor. **a** Schematic of the chiral gold nanoparticle array used as a medium to distinguish LCP light and RCP light. **b** Circular dichroism and extinction data of the chiral gold nanoparticle array. **c** Schematic of the CPL detector consisting of a chiral gold nanoparticle array integrated with an InGaZnO transistor that acts as a hot electron acceptor. **d** Corresponding energy band diagram of the CPL-detecting transistor.

The device schematic of the CPL detector using chiral gold nanoparticles is presented in Fig. 1a, and the IGZO/chiral gold nanoparticle structure is expected to distinguish LCP and RCP light. A chiral gold nanoparticle array in polydimethylsiloxane (PDMS) was fabricated (Fig. S1 and Methods), and the CD data of the chiral gold nanoparticle array show a strong chiroptical response, as shown in Fig. 1b (CD ∝ A_LCP_-A_RCP_). Based on our previous discovery, the strong CD signal ranging over a few degrees is largely attributed to the highly twisted chiral structure created during peptide-directed chemical synthesis^16^. The CD data show two distinct opposite peaks at ~638 nm and ~789 nm, in which a negative value means higher absorption of RCP light, while a positive value means higher absorption of LCP light.

The CPL-detecting transistor is fabricated by forming a Schottky contact between the chiral gold nanoparticles and the IGZO transistor, and the photocurrent is measured at the drain electrode under CPL illumination. (Fig. 1c) The corresponding energy band diagram of the device is presented in Fig. 1d, in which the Schottky barrier is determined by the difference between the work function of the metal particle and the electron affinity of the semiconductor. The work function of chiral gold nanoparticles is measured as 4.79 (±0.02) eV using the Kelvin Probe (see Methods for detailed experimental details), and the electron affinity of IGZO is reported as 4.2 eV^17^, resulting in Schottky barrier formation (~0.59 eV). If the energy of the incident light is higher than 0.59 eV, then hot electrons from the chiral gold nanoparticles can jump over the Schottky barrier, giving rise to a measurable photocurrent. The two distinct wavelengths with different signs in CD are selected to investigate whether the photocurrent follows the different absorption trends shown in the CD graph. Both 635 nm and 780 nm are selected, and the photocurrent contributed by the IGZO layer is eliminated since the energy of incident light is less than the bandgap of the IGZO layer (3.2 eV)^18^. Moreover, the IGZO layer does not contribute to the CD (Fig. S2).

**Fig. 2:**
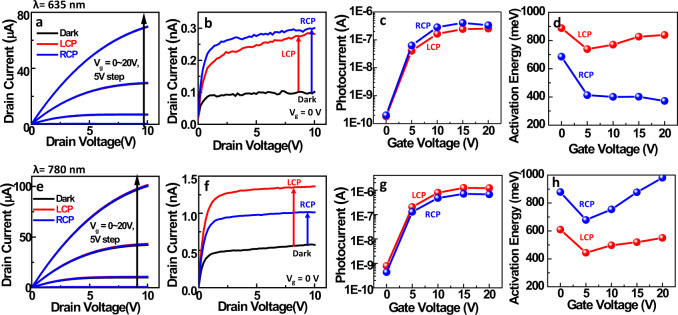
Photocurrent and activation energy of the IGZO-chiral gold nanoparticle array. **a**, **e** Output curve of the CPL-detecting transistor under illumination with 635 nm and 780 nm CPL, respectively. The power density of LCP and RCP light is precisely calibrated at 3.7 mW/cm^2^, and the active area is 100 μm $\times$ 50 μm. **b**, **f** Magnified output curve when the gate voltage is zero. **c**, **g** Photocurrent with respect to gate voltage under 635 nm and 780 nm CPL illumination, respectively. **d**, **h** Activation energy depending on the gate voltage under LCP and RCP light illumination at 635 nm and 780 nm wavelengths, respectively.

In the device configuration, the output curve under CPL illumination is measured at the two different wavelengths of 635 nm and 780 nm (Fig. 2a, e). The magnified output curve at zero gate voltage is presented in Fig. 2b, f. At the 635 nm wavelength, the output curve shows a higher photocurrent under RCP illumination. In contrast, a higher photocurrent is observed from LCP light at the 780 nm wavelength. It is noticeable that this trend is consistent with previously explained CD data. The photocurrent at a drain voltage of 10 V is extracted according to the gate voltage (Fig. 2c, g). The photocurrent increases as the gate voltage increases regardless of the wavelength or circular polarization state. The increased photocurrent may come from accelerated migration of hot electrons caused by the gate voltage^13^. The transfer curve of the CPL detector under CPL illumination at the two different wavelengths is also presented in Fig. S3. At 635 nm, RCP excitation gives rise to a higher subthreshold swing, while a higher subthreshold swing is observed under LCP excitation at 780 nm. By combining the photocurrent and subthreshold swing, the CPL detector may further be utilized to simultaneously multiplex the circular polarization state and incident light wavelength. Different handedness of the chiral nanoparticles displays the opposite trend of CD and device characteristics, (Fig. S4) and such result proves that the chiral nanoparticle plays a critical role for CPL sensing.

The photoresponsivity of the device is calculated as 1.37 A/W and 1.81 A/W at a gate voltage of 20 V under 635 nm LCP and RCP light illumination, while the device shows a photoresponsivity of 6.5 A/W and 3.62 A/W at a gate voltage of 20 V under 780 nm LCP and RCP light illumination. To the best of our knowledge, such high photoresponsivity in the visible light range has not been achieved in previous studies. Table 1 shows the photoresponsivity of previously reported hot electron photodetectors, and our device shows about 1000 times higher photoresponsivity compared to the only reported near infrared (NIR) operating CPL detector^9^. Moreover, our device still shows higher photoresponsivity compared to linearly polarized light detectors^19–24^. The number of hot electrons contributing to the photocurrent is quantitatively calculated, and it is found that a single chiral gold nanoparticle gives rise to the injection of 8.4 $\times$ 10^5^ hot electrons, and hot electron contribution efficiency is about 4% under 780 nm LCP excitation with gate voltage of 20 V (Supplementary information and Fig. S5). Another figure of merits of photodetectors such as the linear dynamic range (LDR), specific detectivity, and −3 dB frequency are also investigated. The photocurrent is measured as a function of light intensity, and the LDR is calculated as 53.06 dB (Fig. S6). The specific detectivity is calculated as ~8 $\times$ 10^9^ Jones and ~2 $\times$ 10^10^ Jones at 635 nm and 780 nm wavelength, respectively (Fig. S7). The light modulation frequency is controlled, and the frequency that causes −3 dB attenuation is measured over 100 Hz without gate voltage, and a lower frequency is measured with the gate voltage (Fig. S8). The −3 dB frequency at a zero gate voltage is high enough for imaging application (>30 Hz).

**Table 1:** Previously reported hot electron-based photodetectors

Light type	Range	Wavelength	Device structure	Photoresponsivity		Ref
Circularly Polarized Light	NIR	1.35 μm	Chiral Ag/ Si	~4.3 mA/W (LCP)	~1.5 mA/W (RCP)	^9^
	Visible	780 nm	Chiral Au NP/IGZO Transistor	4.26 mA/W (V_g_ = 0 V, LCP) **6.50** **A/W** **(V**_**g**_ **=** **20** **V, LCP)**	2.37 mA/W (V_g_ = 0 V, RCP) **3.62** **A/W** **(V**_**g**_ **=** **20** **V, RCP)**	**Our Works**
		635 nm		0.98 mA/W (V_g_ = 0 V, LCP) **1.37** **A/W** **(V**_**g**_ **=** **20** **V, LCP)**	1.08 mA/W (V_g_ = 0 V, RCP) **1.81** **A/W** **(V**_**g**_ **=** **20** **V, RCP)**	**Our Works**
Linearly Polarized Light		1.31, 1.55 μm	Au/n-Si	13.3 mA/W		^19^
	NIR	1.55 μm	Au/graphene/n-Si	85 mA/W(V = 0.1 V) 0.37 A/W (V = 3 V)		^20^
		1.2–1.8 μm	Au,Pd/p-Si	1 A/W		^21^
		400–750 nm	Au NP/TiO_2_	9 μA/W		^22^
	Visible	400–900 nm	Au/TiO_2_	70 mA/W		^23^
		450–700 nm	Au NP/ZnO Transistor	0.14 A/W(V_g_ = 0 V) ~3 A/W(V_g_ = 20 V)		^24^

To understand the different photocurrent levels with regard to the circular polarization state, an Arrhenius plot is analyzed to calculate the activation energy. The photocurrent with varying temperature is measured under CPL illumination at the two different wavelengths (Fig. S9). By drawing a linear fit in the Arrhenius plot, each activation energy is calculated. The extracted activation energy under CPL illumination at the two different wavelengths is presented according to the gate voltage (Fig. 2d, h). In detail, the activation energy under RCP light illumination is lower than that under LCP light illumination at the 635 nm wavelength (Fig. 2d). In contrast, the opposite trend is observed at 780 nm (Fig. 2h). The trend in the activation energy according to the circular polarization state is exactly the opposite to the trend in the photocurrent. Accordingly, we can assume that a low activation energy gives rise to a high photocurrent. It is meaningful that the activation energy is differentiated by the circular polarization state under the same device structure and the same Schottky barrier height. The origins of the different activation energies depending on the circular polarization state can be attributed to hot electron generation and hot electron transport over the Schottky barrier. In terms of hot electron generation, the numbers of hot electrons generated by LCP and RCP light can be differentiated based on CD data. At the 635 nm wavelength, RCP illumination can result in more hot electrons than LCP illumination due to the higher absorption of RCP light. In contrast, at the 780 nm wavelength, LCP illumination can result in an increased number of hot electrons compared to RCP illumination. The calculated activation energy under 635 nm CPL illumination is ~890 meV (LCP) and ~690 meV (RCP) at zero gate voltage, and it decreases when a gate voltage exists (Fig. 2d). Under 780 nm CPL illumination, the calculated activation energy is ~610 meV (LCP) and ~880 meV (RCP) at zero gate voltage, and it decreases when a gate voltage exists (Fig. 2h). Without light illumination, the activation energy is calculated as ~257 meV at a zero gate voltage (Fig. S10) and it is attributed to the Schottky barrier from IGZO-molybdenum (Mo) contact. At zero gate voltage, it is noticeable that the calculated activation energy under light illumination is similar to the sum of two Schottky barriers of chiral gold nanoparticle-IGZO contact and IGZO-Mo contact. Moreover, an increased number of hot electrons can even reduce the activation energy at the same Schottky barrier, and this trend is consistently observed under various light intensity conditions (Fig. S11). The decreased activation energy at a positive gate voltage may come from the accelerated hot electron injection and quantum tunneling effect enabled by the gate voltage^24^. The detailed hot electron generation and transport in our system will be discussed in Figs. 4 and 5.

**Fig. 3:**
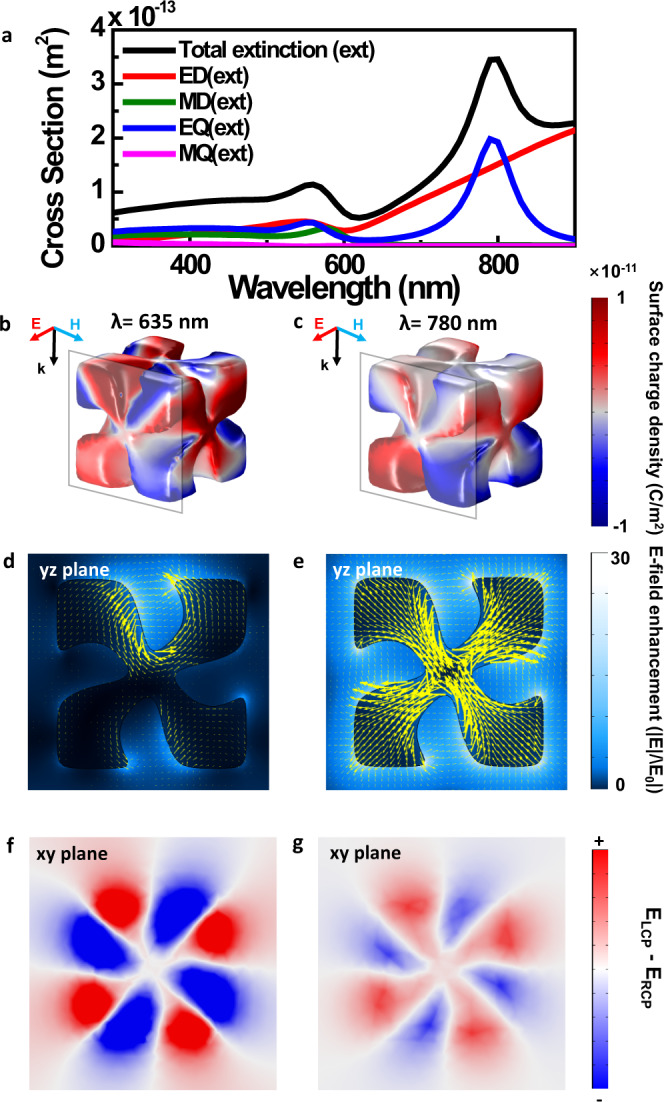
Simulation analysis of a chiral gold nanoparticle and the IGZO/chiral gold nanoparticle interface. **a** Multipole expansion of the total extinction of a single chiral gold nanoparticle of 180 nm. **b**, **c** Surface charge distribution under linear excitation (x-polarization) with 635 nm and 780 nm, respectively. **d**, **e** E-field enhancement and its corresponding current distribution in the yz plane (yellow arrow) at x = −80 nm under linear excitation (x-polarization) with 635 nm and 780 nm, respectively. **f**, **g** Difference between the E-field enhancement under LCP and RCP excitation in the xy plane, which is the interface between the chiral gold nanoparticle and IGZO under 635 nm and 780 nm light excitation, respectively. The integrated value is −1.20 $\times$ 10^−15^ m^2^ (635 nm) and 7.10 $\times$ 10^−15^ m^2^ (780 nm).

The photocurrent trend can be corroborated by simulation data. To analyze the difference in the photocurrent according to the wavelength, the individual multipole contribution of the total extinction of a single chiral nanoparticle was calculated (Fig. 3a). It is confirmed that stronger extinction is observed at ~800 nm than at ~600 nm, in which extinction near 600 nm is attributed to the electric dipole, magnetic dipole and electric quadrupole moments, while extinction near 800 nm is attributed to the electric dipole and electric quadrupole moments. This simulation is related to the hot electron generation in chiral gold nanoparticles; however, it cannot fully represent the photocurrent, since only a small portion of hot electrons actually transported near the interface between chiral gold nanoparticles and the semiconductor can contribute to the photocurrent. Therefore, the surface charge distribution data and the electric field enhancement(|E/E_0_|) in the yz plane (Fig. 3b–e) are investigated at two specific wavelengths (635 nm and 780 nm). Higher excitement of the rear part of the particle at 780 nm is observed, and it implies a greater possibility of hot electron transport into the semiconductor. Therefore, this simulation comprehensively explains the higher photocurrent at 780 nm than at 635 nm.

To analyze the distinct chiroptical responses of the chiral gold nanoparticle, photonic simulations were conducted with the IGZO/chiral gold nanoparticle structure under illumination by wavelength-swept LCP and RCP light. The difference in the E-field enhancement between LCP and RCP light (|E_LCP_|-|E_RCP_|) at the xy plane that is the interface between the chiral gold nanoparticle and IGZO is presented in Fig. 3f, g, in which a negative area is broadly observed under 635 nm light excitation (Fig. 3f), and vice versa under 780 nm light excitation (Fig. 3g). It is noticeable that the simulation data can consistently explain the degree of photocurrent depending on the wavelength and circular polarization state. Therefore, three-dimensional chiral plasmonic materials can successfully act as wavelength- and circular polarization state-dependent filters.

It is known that hot electron generation is accelerated at hot spots in plasmonic nanostructures^25,26^, in which the E-field is significantly enhanced due to light confinement in the nanostructures. Accordingly, hot electron generation of chiral plasmonic particles can also be explained by circular polarization state dependent E-field enhancement; however, hot electrons are difficult to experimentally prove due to their fast relaxation. Herein, we try to prove the difference in the amounts of hot electrons generated depending on the circular polarization state through femtosecond laser pump-probe spectroscopy. Based on the strong chiroptical response of the chiral particle array (Fig. 1b), one can expect that plasmonic excitations with LCP and RCP beams would lead to significantly different hot carrier generation and dynamics in the chiral gold nanoparticle, which eventually modulate the generation of a photocurrent via hot electron injection into the semiconductor. To investigate the plasmon-induced hot carrier dynamics and their connection to the different photocurrent generation under LCP and RCP illumination, we performed a polarization-dependent transient absorption (TA) experiment with LCP and RCP pump beams (Fig. S12).

**Fig. 4:**
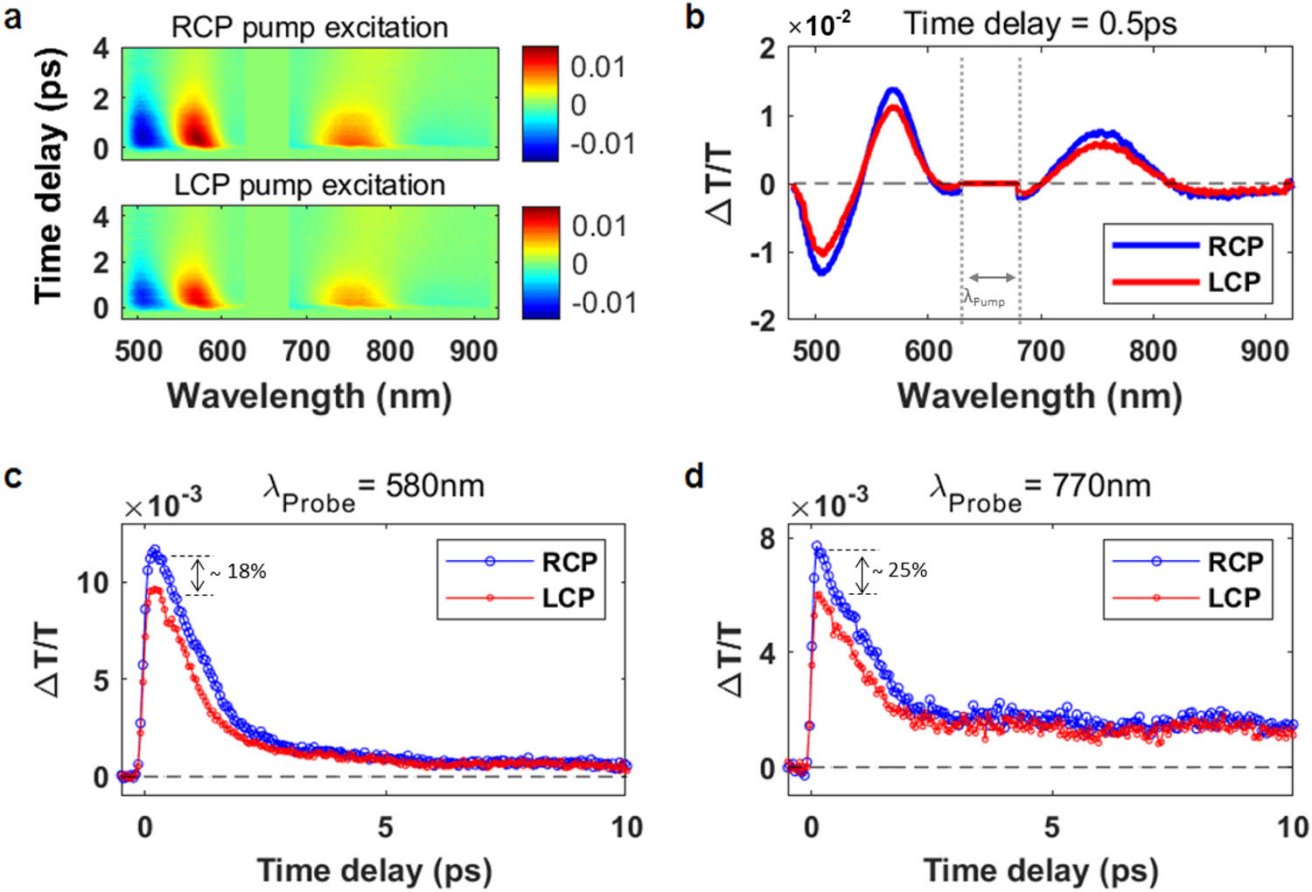
Polarization-dependent broadband transient absorption (TA) dynamics in chiral gold nanoparticle arrays. **a** Time-resolved TA (ΔT/T) surface maps of the chiral gold nanoparticle array measured with RCP (top) and LCP (bottom) pump beams. **b** TA spectral cut taken at a pump-probe time delay of 0.5 ps under RCP (blue) and LCP (red) pump illumination. Note that the TA signal values in the range of 630–680 nm (between the dashed lines), where strong pump scattering noise (λ_pump_ = 650 nm) interferes with the weak TA signal, are intentionally set to zero for a better view of the TA signals in other spectral regions of interest. **c**, **d** Photobleaching (PB) traces as a function of pump-probe time delay with RCP (blue) and LCP (red) pump beams at 580 nm and 770 nm, respectively.

Figure 4a shows time-resolved TA surface maps of a chiral gold nanoparticle after femtosecond plasmonic excitation at 650 nm with LCP and RCP pump beams. At early times (pump-probe time delay: τ < 2 ps), two significant photobleaching signals (PB, ΔT/T > 0) are observed around the plasmon band maximum wavelengths (560 nm and 780 nm), while photoinduced absorption signals (PA, ΔT/T < 0) appear at both sides of each PB signal peak, as seen in Fig. 4b. These are similar to typical spectral features often observed in the TA spectra of plasmonic metal nanoparticles^27–30^ and can be explained by a change in the dielectric constant due to electron heating (hot electron generation)^28,29^. The energetic electrons excited above the Fermi energy level by an intraband transition undergo rapid electron thermalization, which elevates the electronic temperature, via electron-electron collisions (scatterings) within 0.1–1 ps^31,32^. The increased electronic temperature due to electron heating significantly changes the dielectric constant of the gold nanoparticle. The main effect of such a change is a spectral broadening of the plasmon band, which leads to a strong PB signal near the plasmon band maximum and PA signals in the wings of the band^27–30^. These features are in good agreement with the observed TA data in Fig. 4a and b.

A larger spectral broadening resulting from a more increased electronic temperature produces a larger amplitude of the PB or PA signal, as explained above. The peak amplitude of a PB or PA signal is thus a measure of the hot electron population. To examine the dependence of hot carrier generation on the handedness of the CPL pump beam, the PB traces with LCP and RCP beams at 580 nm and 770 nm are plotted in Fig. 4c and d for comparison. The PB signals show a fast rise within τ = 500 fs due to electron heating and a subsequent decay with a time constant of 0.93 ps–1.1 ps due to electron-phonon energy exchange^28,32,33^. This result shows the common feature of their transient dynamics. However, there is a notable difference in the peak amplitudes. The RCP excitation induces a larger PB signal amplitude than the LCP excitation does. For example, the PB signals for RCP excitation at τ = 500 fs exhibit larger amplitudes than those for LCP excitation by 18% and 25% at 580 nm and 770 nm, respectively. This observation indicates that the RCP pump beam creates greater hot carrier populations in the chiral gold nanoparticle than the LCP pump beam does. This TA result is in good agreement with the steady-state CD (Fig. 1b) data having a negative CD value near the higher energy plasmon band (635 nm) and supports the fact that the hot electron population serves as the primary source of the differential photocurrent generation (Fig. 2) in the chiral gold nanoparticle under plasmonic excitation with LCP and RCP beams.

**Fig. 5:**
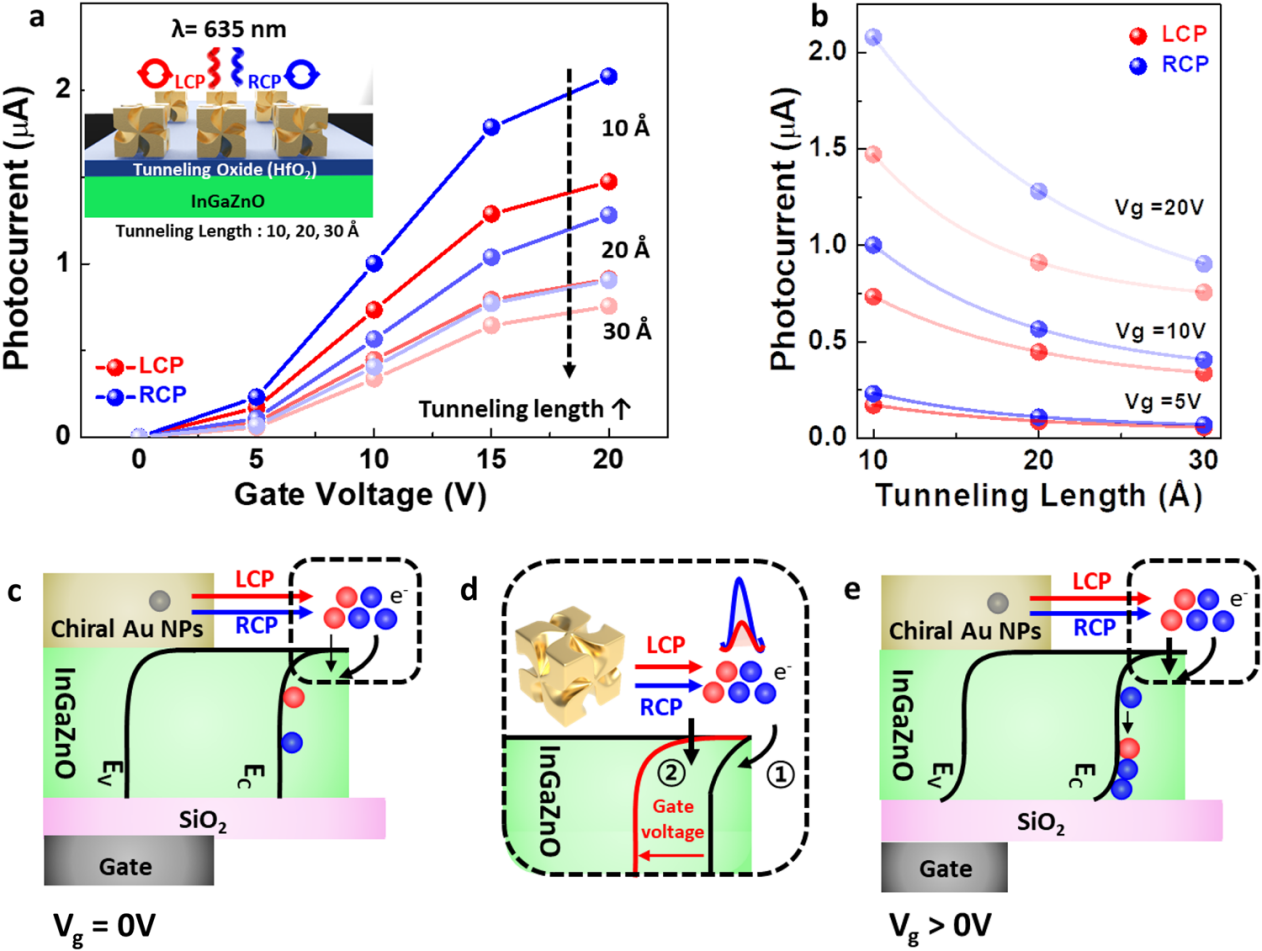
Photocurrent of the CPL-detecting transistor with a tunneling oxide, and suggested hot electron transport mechanism depending on the gate voltage. **a** Photocurrent of the CPL-detecting transistor under 635 nm CPL illumination when the device has a tunneling HfO_2_ film of various thickness at the interface between the chiral gold nanoparticles and InGaZnO layer. **b** Corresponding photocurrent with respect to tunneling length, and fit to an exponential decay. **c–e** Hot electron migration schematics when the gate voltage is zero (**c**) and positive (**e**). Magnified energy band bending of the Schottky barrier depending on the gate voltage-dependent InGaZnO energy band bending of the CPL-detecting transistor (**d**).

The excited hot electrons having higher energy than the Schottky barrier are known to be injected into the semiconductor^7,8,34^, and hot electrons having lower energy than the barrier can also give rise to a small tunneling current^35^. In addition, it was discovered that the gate voltage of the transistor could tailor the slope of the Schottky barrier bending, resulting in acceleration of both the thermionic and tunneling currents^24^. To confirm the hot electron tunneling mechanism in our device, the photocurrent with respect to the thickness of the tunneling oxides was measured. Tunneling oxides were deposited through an atomic layer deposition (ALD) process, and oxides of different lengths (10–30 Å) were inserted at the interface between the chiral gold nanoparticles and IGZO semiconductor. The governing equation of the tunneling current is expressed by the multiplication of the electron number and transmission probability^36,37^, in which the probability exponentially decreases with increasing tunneling barrier length (T ~ e^−2γL^, where T is the transmission probability of electrons, γ is the wavenumber or absorption coefficient inside the barrier and L is the barrier length.)^38,39^. As a result, the photocurrent decreases with increasing tunneling oxide length, and under the same tunneling oxide length, RCP excitation at 635 nm gives rise to a higher photocurrent. (Fig. 5a, Fig. S13) The photocurrent is well fitted to an exponential decay curve, (Fig. 5b, Fig. S13) and we can conclude that both a quantum tunneling process and thermionic process contribute to hot electron transport in our device. Under the tunneling current equation, different hot electron populations depending on the circular polarization state can also contribute to an increase in the tunneling current. Accordingly, different numbers of hot electrons depending on the circular polarization are injected through the thermionic process into the IGZO semiconductor without a gate voltage (Fig. 5c). With a gate voltage, hot electrons can be injected by both thermionic and tunneling processes, and the injected hot electrons can accumulate at the SiO_2_ interface (Fig. 5e). The corresponding slope of the Schottky barrier is drastically bent by the gate voltage (Fig. 5d), and the photocurrent is significantly enhanced by the gate voltage (Fig. 2).

The previously measured activation energy (Fig. 2) can be explained by hot electron generation and transport. Regardless of the gate voltage, the different numbers of hot electrons depending on the circular polarization state have an impact on the activation energy. On the other hand, the gate voltage-induced quantum tunneling process seems to decrease the activation energy (Fig. 2d). There is one previous study covering the activation energy calculation for hot electron-based photocatalysts depending on the plasmonic wavelength and power density of light^40^. Although the activation energy previously reported does not come from the Schottky barrier but from the NH_3_ decomposition process, the calculation is important for designing energy-efficient plasmonic photocatalysts. In our system, the quantitative calculation of the activation energy and well-explained trends in the hot electron population and transport over the Schottky barrier are also meaningful for designing a high-performance CPL detector using a three-dimensional plasmonic chiral structure.

**Fig. 6:**
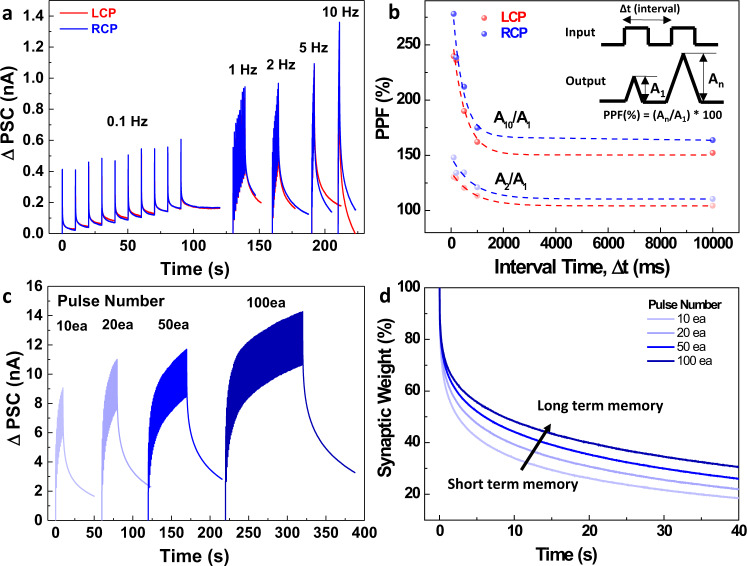
Synaptic Characteristics of CPL-Detecting Transistor. **a** Delta post synaptic current under CPL pulse illumination at 635 nm depending on frequency. The light intensity was set to 4.9 mW (**b**) PPF(paired pulse facilitation) as a function of pulse interval time. The graph is fitted into double exponential function: y = A_1_ exp(−t/τ_1_), + A_2_ exp(−t/ τ_2_), where t is the interval time. **c** Delta post synaptic current and (**d**) synaptic weight trend after light is turned off according to CPL pulse number.

The applications of CPL detector can be diverse if the device can perform both detecting and memorizing. Such device can perform image classification work and it can significantly reduce latency and power consumption of the system such as imaging and LiDAR application. Therefore, we further investigated light pulse dependent photocurrent characteristics of the CPL-detecting transistor. The CPL pulses at 635 nm with different operating frequency are utilized as an input of the CPL detector and the delta post synaptic current (PSC) is measured. (Fig. 6a) The current is enhanced as the light pulse number increases, and such facilitating characteristics is fundamental for the memory and learning process in the synapse. As the light frequency rises, the current increases and higher current is observed when RCP light pulses are illuminated. Paired pulse facilitation (PPF) is one of the basic synaptic characteristics, and the value is calculated by A_n_/A_1_ $\times$ 100% where A_n_ is the intensity of current when *n*th pulse is illuminated. As shown in Fig. 6b, PPF increases as the pulse time interval decreases and the trend is well fitted to exponential decay, which is general learning model of neurons. In detail, A_10_/A_1_ shows higher PPF than A_2_/A_1_, and PPF calculated by RCP light shows higher value than that by LCP light due to chiroptic response of chiral gold nanoparticle.

The relaxation characteristics after the light turns off, is important for the memorizing and classification work, therefore, relaxation of PSC is investigated as the number of light pulses varies. As shown in Fig. 6c, the synaptic current increases as the number of RCP light pulse increases. The Fig. 6d shows that the synaptic weight remains more when the number of light pulses is more, and such change represents that short-term plasticity is transformed into long-term plasticity (LTP) by repeated pulse stimulation. That is, the plasticity of the device is controlled by the number of light pulses, and it resembles memory consolidation process suggested by Atkinson and Shiffrin^41^.

**Fig. 7:**
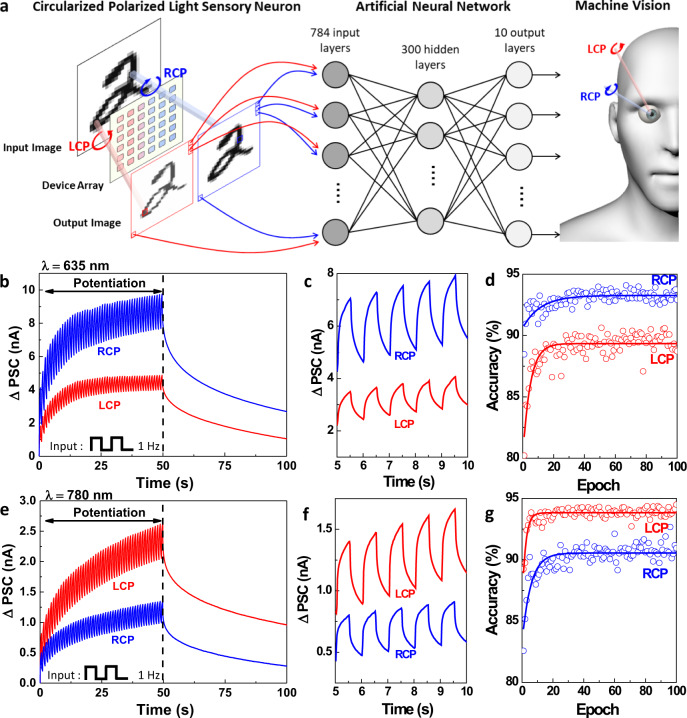
Polarization Dependent Image Classification. **a** Artificial neural network structure based on circularly polarized light sensory neuron to perform handwritten digit classification work using the MNIST database, and schematic of machine vision featuring differences in learning efficiency depending on the circular polarization state. **b** Potentiation curve under 1 Hz CPL pulse illumination at a 635 nm wavelength, and decaying curve without light illumination. The intensity of CPL was set to 4.9 mW (**c**), Magnified post synaptic current between 5.0 and 10.0 s, in which the current well responds to the 1 Hz CPL pulse input. **d** Handwritten digit classification simulation results based on potentiation curve at 635 nm LCP and RCP light pulses. **e** Potentiation curve under 1 Hz CPL pulse illumination at a 780 nm wavelength, and decaying curve without light illumination. **f** Magnified post synaptic current between 5.0 and 10.0 s, in which the current well responds to the 1 Hz CPL pulse input. **g** Handwritten digit classification simulation results based on the potentiation curve at 780 nm LCP and RCP light pulse.

We further investigated the function of CPL detector as a sensory neuron, which features current accumulation according to the number of presynaptic spikes using LTP condition. The CPL pulses (1 Hz, duty cycle: 50%) at 635 nm and 780 nm are utilized as an input of the CPL detector and the delta PSC is measured. As shown in Fig. 7b and e, PSC increases as the light pulse number increases, and a different degree of current accumulation is observed depending on circular polarization state. Under 635 nm light pulse training, higher PSC is observed under RCP illumination (Fig. 7c), while the opposite trend is obtained under 780 nm CPL pulse training (Fig. 7f). After applying the final CPL pulse, ~4.87 nA (LCP) and ~9.75 nA (RCP) of PSC are observed at a 635 nm wavelength, and ~2.63 nA (LCP) and ~1.35 nA (RCP) of PSC are observed at a 780 nm wavelength.

The PSC curves are measured at V_g_ = 200 mV and V_d_ = 10 V, and the accumulation effect, the key to synaptic training, is enabled by applying a moderate gate voltage. The CPL pulses create hot electrons in chiral gold nanoparticles, and gate voltage facilitates the accumulation of hot electrons at the interface between IGZO and the dielectric layer (SiO_2_), resulting in the potentiation curve. Applying different signs and degrees of gate voltages gives rise to difference in hot electron accumulation and hot electron trapping at the interface, and various potentiation and relaxation curves can be achieved by finely controlling the gate voltage. (Fig. S14) Regarding relaxation curve, minus gate voltage shows high remaining synaptic weight, (Fig. S15) and it implies that hot electrons remain well at the interface due to electrostatic interaction (Fig. S16) The origins for such synaptic current are different from those suggested in previous IGZO-based opto-neuromorphic applications; ultraviolet light-directed persistent photoconductivity of IGZO^42^ and hole trapping at the interface between IGZO and dielectric material^43^. Since the energy of light is far lower than the bandgap of IGZO in our system, hot electrons from chiral gold nanoparticles are considered a key factor in differences in post-synaptic current accumulation and relaxation with respect to the circular polarization state.

We also confirmed the feasibility of the devices for neuromorphic application using ANN simulation (Fig. 7a). The fully connected neural network with three neuron layers is trained with the backpropagation algorithm to perform a classification task on the Modified National Institute of Standards and Technology (MNIST) data set of handwritten digits. The Gaussian noise is intentionally added into MNIST database to certify noise reduction effect in our CPL sensory neurons depending on the circular polarization state (see Methods section). Figure 7d shows the classification simulation results, where the accuracy increases with increasing training epochs regardless of circular polarization states. At a 635 nm wavelength, higher accuracy is observed when the RCP light pulse-trained curve is used, and this trend is consistent with that of the potentiation curve. In contrast, the opposite trend is observed at a 780 nm wavelength as shown in Fig. 7g. In the final training epochs, the accuracy of the CPL detector-based ANN reaches ~89.3% (LCP), ~93.3% (RCP) under 635 nm light, and ~93.8% (LCP), ~90.5% (RCP) at a 780 nm wavelength (Fig. 7d, g). As a result, the CPL-detecting transistor can be applied to hardware implementation of artificial sensory neuron that can finely control the learning efficiency depending on the circular polarization state, and we believe that polarization control enabled by chiral plasmonic nanoparticle can expand the degrees of freedom in the field of in-sensor computing machine vision applied to LiDAR^44^ for material identification and biomedical imaging^45^ for early diagnosis of cancer. Since CPL could significantly improve reliability of LiDAR even at bad weather conditions, and have long penetration length within biological tissue of human in biomedical imaging, circular polarization dependent opto-neuromorphic operation could be versatile used. Moreover, such adaptive learning in accordance with the environment is a key biological feature^46^, and the demonstration of the CPL-dependent learning device will also facilitate realization of the multimodal and cooperative activation of a neuromorphic device^47^ that resembles biological synapse in a more sophisticated way.

In summary, our study demonstrates visible light operating hot electron-based CPL detector with high photoresponsivity. The superior performance is attributed to the strong chiroptical response of the chiral gold nanoparticles and acceleration of hot electron injection by IGZO transistor. This observation is explained by quantitatively calculated activation energy, simulation data, hot electron generation, and hot electron transport mechanism. Noticeably, different hot electron populations are experimentally proven by polarization-dependent femtosecond pump-probe spectroscopy. We expect that our study not only broadens the fundamental understanding of hot electron generation in chiral plasmonic metamaterials but also paves the way for practical compact polarimeters and machine vision system in integrated chips.

## Methods

### Fabrication of chiral gold nanoparticle array

In a typical gold nanoparticle synthesis, a growth solution was prepared by adding 0.8 ml of 100 mM Hexadecyltrimethylammonium bromide (CTAB, 99%, Sigma Aldrich) and 0.2 ml of 10 mM tetrachloroauric(iii) trihydrate (HAuCl_4_·3H_2_O, 99.9%, Sigma Aldrich) into 3.95 ml of deionized water to form an [AuBr_4_]^−^ complex. Au^3+^ was then reduced to Au+ by the rapid injection of 0.475 ml of 100 mM L-ascorbic acid (AA, 99%, Sigma Aldrich) solution.

The growth of chiral nanoparticles was initiated by adding 5 μl of peptide solution and 50 μl of seed solution into the growth solution. To prepare chiral gold nanoparticle, 5 mM glutathione (γ-E-C-G, 98%, Sigma-Aldrich) was added to the growth solution, followed by the addition of octahedral seed solution, in which the octahedral seed was synthesized as reported previously^48^, and dispersed in aqueous CTAB (1 mM) solution. The growth solution was placed in a 30 °C bath for 2 h, and the pink solution gradually became blue with large scattering. The solution was centrifuged twice (1,677 g, 60 s) to remove unreacted reagents and was re-dispersed in a 1 mM CTAB solution for further characterization.

To fabricate chiral gold nanoparticle array, PDMS was patterned by soft lithography using pillar shaped silicon mold featuring regularly separating distance of 400 nm. Synthesized chiral gold nanoparticles are coated on pre-patterned PDMS, and the chiral particles were inserted into regularly separated holes by mechanical rolling.

### Circular dichroism analysis

For the CD analysis, chiral particle array was placed on quartz substrate. The CD spectrum of the chiral gold particle array was recorded using spectropolarimeter (J-1500, Jasco) at 25 °C. The spectrum was collected from 900 to 300 nm with 1 nm interval keeping the HT voltage <600 V for reliability.

### Kelvin probe analysis

The work function of chiral gold nanoparticles was measured using a scanning Kelvin probe (SKP5050, Kelvin Probe Technology, U.K.), which measures the surface electrical potential without contacting the sample. The work function was calculated from the potential difference between the chiral gold particles and the Au tip, for which the work function value is already known.

### Fabrication procedure of InGaZnO transistor

50 nm-thick IGZO semiconducting layer was deposited by radio frequency (RF) sputtering on SiO_2_/Si substrate. The sputtering was performed at room temperature with a base pressure of <10^−6^ torr, a power of 100 W and gas flow rates of 30 s.c.c.m. and 0.5 s.c.c.m. for Ar and O_2_, respectively. The deposited area of 500 µm $\times$ 1000 µm was patterned by standard photolithography. Then, 100 nm-thick Mo was deposited by direct current (DC) sputtering with a power of 200 W and Ar gas flow rate of 30 s.c.c.m., and patterned by standard photolithography. The width and length of the device are defined as 100 µm and 50 µm, respectively. To explain hot electron injection process (Fig. 5), tunneling barrier of HfO_2_ was additionally deposited by ALD at 150 °C.

### Photocurrent measurement

Photocurrent at IGZO/chiral gold nanoparticle photodetector was measured when the device was illuminated with CPL at normal incident angle. Photocurrent was calculated by subtracting current measured without light illumination from that with light illumination. The output curve and transfer curve were inspected using semiconductor characterization system (Keithley SCS 4200). The linearly polarized light was created using temperature-controlled laser diode mount (Thorlabs, TCLDM9), and lasing wavelength is tailored by mounting different laser diodes having characteristic wavelength at 635 nm (Thorlabs, HL5322G) and 780 nm (Thorlabs, L780P010). Circularly polarized light (CPL) was precisely generated with a manually aligned quarter-wave plate (Thorlabs, AQWP05M-600), and ideality of circular polarization was confirmed by commercial polarimeter (Thorlabs, PAN5710VIS). The power of incident light was measured by power meter (Newport, 1918-R), and the intensity of LCP and RCP light is precisely calibrated to calculate photoresponsivity.

### Calculation of figure of merits of the photodetector

The LDR is calculated as *20* *log (I*_*max*_*/I*_*min*_*)*, where *I*_*max*_ and *I*_*min*_ are the maximum and minimum incident light powers within the linear regime, respectively. The specific detectivity (D*) is calculated as ${D}^{*}=\frac{R}{{(2e{I}_{{dark}}/A)}^{1/2}}$, where *R*, *I*_*dark*_ and *A* are the photoresponsivity, dark current and effective areas of the detector, respectively. To obtain −3 dB frequency, the attenuation of response was measured with respect to optical modulation frequency. The function generator (Agilent, 33250 A) was connected to a laser diode controller (Thorlabs, LDC205C), and square voltage pulses give rise to CPL pulses ranging from 0.1 Hz to 100 Hz. The duty cycle was set to 50%, and the light intensity of CPL was checked by the power meter (Newport, 1918-R). Under CPL pulse illumination, the drain current of the device was measured with respect to the light modulation frequency using a semiconductor characterization system (Keithley SCS 4200) in time-stamp mode. It is noted that the highest frequency that we could reliably measure was 100 Hz due to the limit of measuring time resolution in our system. The attenuation of the response was calculated as *20* *log (I*_*1*_*/I*_*0*_*)*, where *I*_*0*_ and *I*_*1*_ are the photocurrent at the initial optical modulation frequency and at the increased optical modulation frequency, respectively. In this way, the attenuation of the response was plotted according to optical frequency, and the frequency that induces attenuation by −3 dB was estimated.

### Simulation analysis

A commercial three-dimensional full-wave Maxwell equation solver (COMSOL) has been used to analyze the optical responses of the chiral nanoparticle and IGZO complex. The calculations were based on the finite element method. The geometry of the simulation model was deduced from SEM images and a triangular mesh was constructed non-uniformly. The reliability of the chiral gold nanoparticle geometry used in the simulation is previously confirmed in our previous literature^16^ by correlating geometry and corresponding optical properties (extinction and CD). The size of chiral gold nanoparticle was 180 nm. The refractive index of PDMS and IGZO was assigned a value of 1.43, 1.95, respectively^49,50^, and the optical properties of gold were taken from Johnson and Christy’s work^51^.

The electromagnetic field near the chiral gold nanoparticle was calculated at a normal incidence toward −z direction. We analyzed the multipolar contribution to the total scattering through multipole decomposition from the calculated electromagnetic field vectors^52^. The surface charge distribution and electric field enhancement(|E|/|E_0_|) were displayed at two different wavelengths (635 nm and 780 nm), where E_0_ indicates an amplitude of the initial electric field. The electric field difference between LCP and RCP excitation ((|E_LCP_|−|E_RCP_|)) is also calculated at the interface between chiral gold nanoparticle and IGZO.

#### Broadband transient absorption measurement system

An Yb:KGW femtosecond amplifier system (Pharos, Light Conversion) centered at 1028 nm with pulse duration of 230 fs was used as a fundamental light source for generating pump and probe beams for broadband TA measurements. An output beam (15 W) from the amplifier system was split into pump and probe paths. One portion was used to pump a non-collinear optical parametric amplifier (NOPA, Light Conversion) to generate 30 fs pump pulses centered at 650 nm. The other portion of the amplifier was focused onto a 4 mm YAG crystal to generate a white light continuum probe beam ranging from 500 to 1100 nm. In order to compensate positive group velocity dispersions (GVD) produced by optical components (YAG crystal, sample cell, etc.), the probe beam was bounced off several times between two negatively chirped mirrors before arriving at the sample. The pump (~28 nJ/pulse) and probe beams were focused onto the sample with a parabolic mirror instead of a focusing lens to minimize the positive GVD of materials. The transmitted probe beam through the sample was spectrally dispersed in a monochromator (SP 2300i, Princeton Instrument) and detected at an electron multiplying charge-coupled device (EMCCD) camera (Newton, Andor). For polarization-dependent TA measurements with LCP and RCP pump beams, the polarization state of the linearly-polarized pump beam was switched between LCP and RCP by using a quarter-wave plate placed in the pump beam path while the probe polarization was set to be linearly polarized. The data acquisition (DAQ) of the EMCCD was triggered by electrical pulses synchronized with the laser repetition rate (250 Hz) while the pump beam was modulated with an optical chopper running at one half of the rate (125 Hz). As a result, the probe spectra with (pump-on) and without (pump-off) the pump beam were alternately recorded at the EMCCD every trigger event, and a TA spectrum (ΔT/T) was then calculated from those pump-on and pump-off spectra at a given pump-probe time delay. Time-resolved TA spectra were obtained by repeating the above DAQ as varying the pump-probe time delay with a motorized linear stage. The phase (sign) of the TA signal (ΔT/T) was automatically corrected by using an additional portion of the probe beam, which is chopped at the same rate with the pump modulation, as a reference clock. The entire DAQ was carried out using a home-built LabView software.

### Simulation of neural networks

The ANN contains 784 input neurons, 300 hidden neurons, and 10 output neurons. For training, all 60,000 images (28 $\times$ 28 pixels) from the MNIST training set are used. The exponentially fitted normalized conductance curves depending on the CPL pulse at 635 nm and 780 nm, are utilized for scaling the input image pixel intensity, and the Gaussian noise (*μ* = 0, *σ* = 0.4) is intentionally added to confirm noise reduction effect depending on the circular polarization state. We updated all the synaptic weights in the ANN by a backpropagation weight updating method. Adaptive moment estimation (ADAM) is used as an optimizer. The batch size and the learning rate are fixed at 128 and 0.01, respectively. In this way, we trained the ANN with 60,000 MNIST training datasets and obtained the recognition rate for 10 numbers between “0” and “9” by examining the trained ANN using 10,000 other testing datasets. The previous literatures are referred to test image classification work^11,12^.

## Supplementary information

Supplementary Information

## Data Availability

The authors declare that the main data supporting the findings of this study are available within the paper and its Supplementary Information files.
