# Supplementary material for: Circularly polarized light-sensitive, hot electron transistor with chiral plasmonic nanoparticles
Source: Nat Commun. 2022 Aug 29;13:5081. doi: 10.1038/s41467-022-32721-2 (PMC9424280; doi:10.1038/s41467-022-32721-2)
Supplement: Supplementary file 1 — Supplementary Information [file 41467_2022_32721_MOESM1_ESM.docx]

**Supplementary Information**

Circularly Polarized Light-Sensitive, Hot Electron Transistor with Chiral Plasmonic Nanoparticles

*Seok Daniel Namgung^1,2^, Ryeong Myeong Kim^1^, Yae-Chan Lim^1^, Jong Woo Lee^1^, Nam Heon Cho^1^, Hyeohn Kim^1^,* *Jin-Suk Huh*^1,2^, *Hanju Rhee^3^, Sanghee Nah^3^,* *Min-Kyu Song^4^, Jang-Yeon Kwon^4^, Ki Tae Nam^1,2^**

1 Department of Materials Science and Engineering, Seoul National University, Seoul 08826, Republic of Korea.

2 Soft Foundry, Seoul National University, Seoul 08826, Republic of Korea

3 Seoul Center, Korea Basic Science Institute, Seoul 02841, Republic of Korea

4 School of Integrated Technology, Yonsei University, Incheon 21983, Republic of Korea

Correspondence to: nkitae@snu.ac.kr (K.T.N.)

**This PDF file includes:**

Figures. S1 to S16


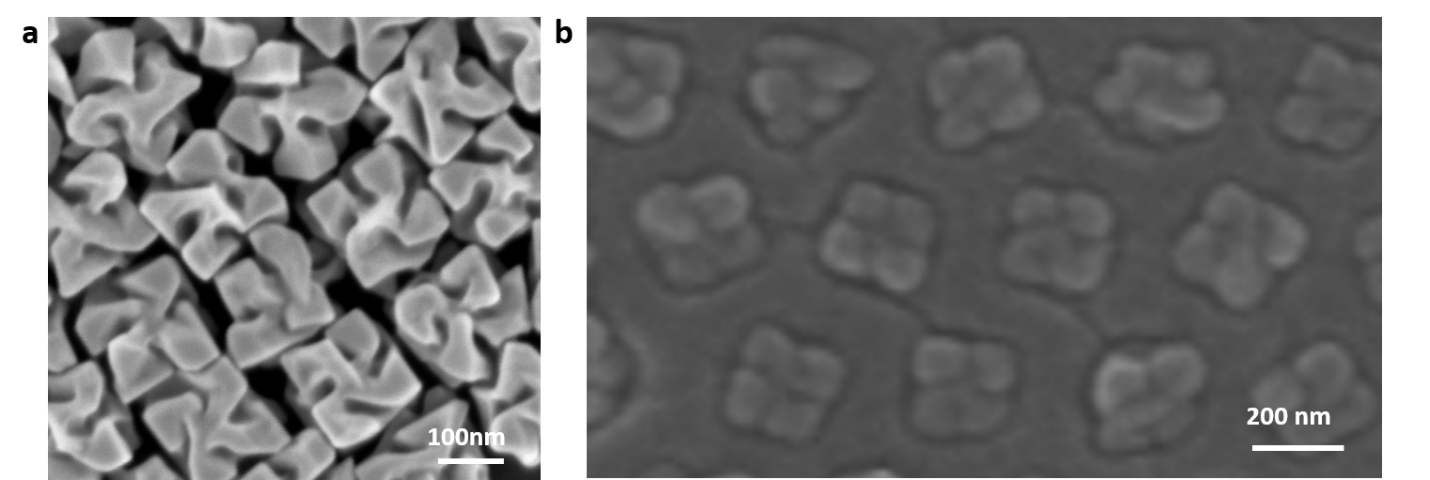


**Figure S1 | a,** SEM image of synthesized chiral gold nanoparticles on a Si substrate, in which the scale bar is 100 nm. **b,** SEM image of chiral gold nanoparticle array in PDMS, in which PDMS was patterned by soft lithography and chiral particles were inserted in regularly separating hole at a distance of 400 nm. The scale bar is 200 nm

**
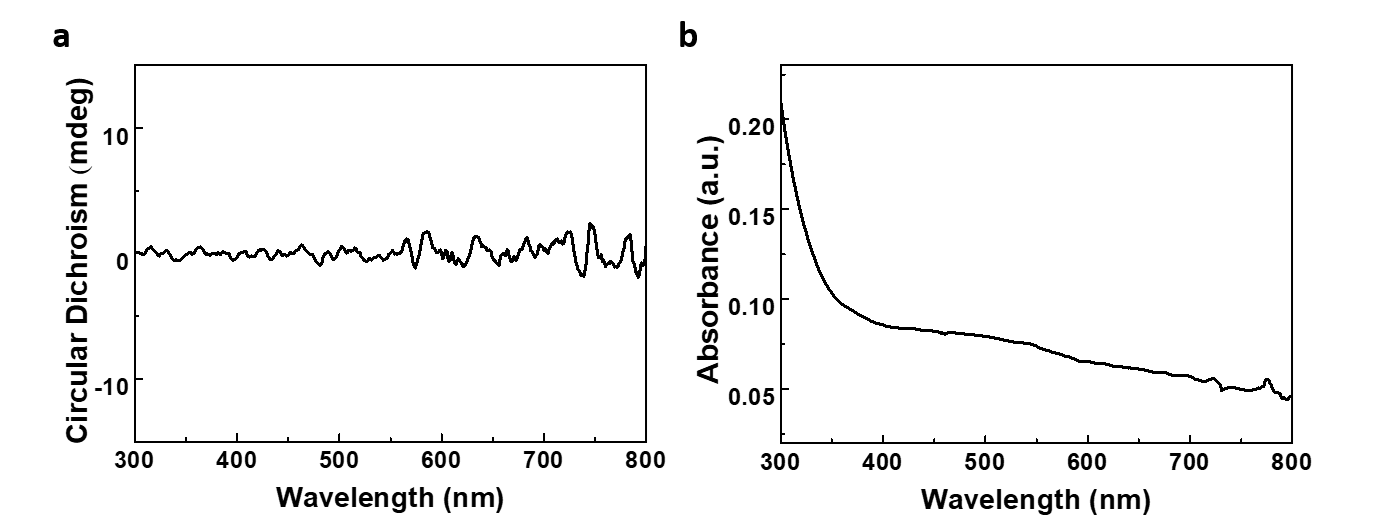
Figure S2 | a,** Circular dichroism of 50 nm thick InGaZnO on quartz sample, in which the sample did not show any circular dichroism from 300 nm to 800 nm. **b,** Absorbance data of the sample, in which, absorbance rapidly increases under ~387 nm wavelength that is correlated with the band gap (3.2 eV) of InGaZnO.


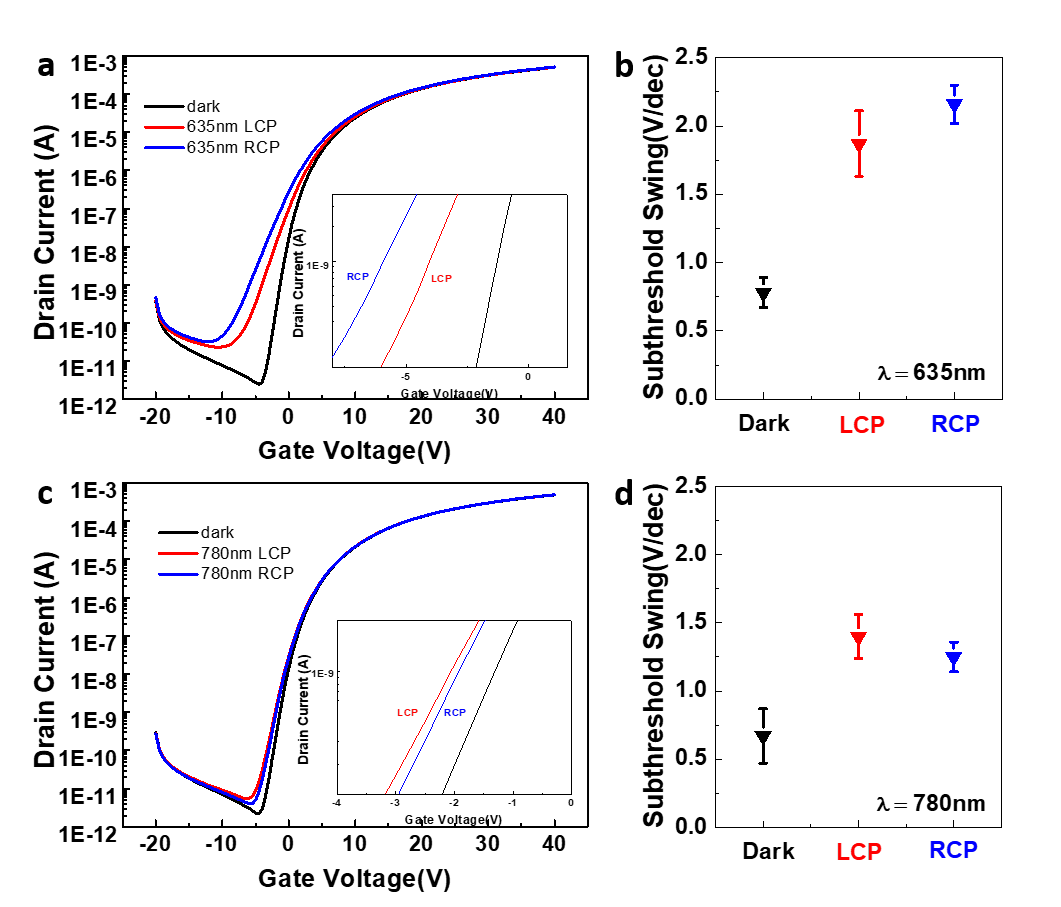


**Figure S3 | a, c,** Transfer curve of IGZO/chiral gold nanoparticle array transistor under 635 nm and 780 nm CPL excitation, respectively. **b, d** Corresponded subthreshold swings (S.S.) of the CPL detector under 635 nm and 780 nm CPL excitation, respectively. At 635 nm wavelength, RCP excitation gives rise to higher S.S.,(b) while higher S.S. is observed at LCP under 780 nm wavelength.(d)


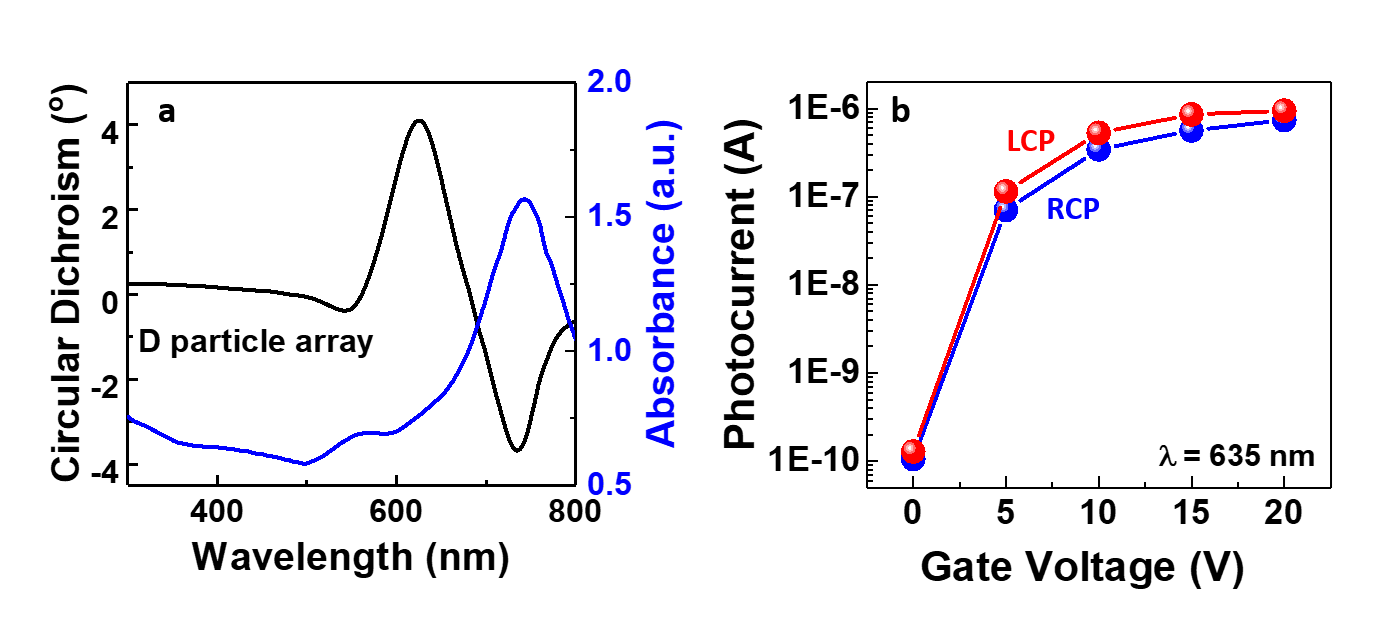


**Figure S4** | **a,** Circular dichroism of D-form chiral nanoparticle array and **b,** photocurrent of the device containing D-form chiral nanoparticles under 635 nm circular polarized light excitation.

**Quantitative calculation on hot electron number**

We calculated the number of hot electrons that contributed to the increase in the drain current. As the transistor device operated in saturation regime, drain current in saturation mode is considered for the calculation. Drain current in saturation mode can be expressed as below.

$$\Delta I_{ds}= \frac{1}{2}\mu_{n}C_{ox}\frac{W}{L}\left( V_{g}-V_{th} \right)^{2}$$

Where C_ox_ is the capacitance per unit area, μ_n_ is the mobility of electrons in the semiconductor layer. V_g_ is the gate voltage and V_th_ is the threshold voltage of the transistor. W and L represent the width and length of the channel region in the device, respectively.

As the transistor is biased at a large voltage condition, small threshold voltages can be ignored. Considering that charge is the product of capacitance and voltage (Q = CV), the increased drain current can be expressed as below.

$$\Delta I_{ds}= \frac{1}{2}\mu_{n}\Delta Q\frac{W}{L}V_{g}$$

The experimentally extracted electron mobility of InGaZnO transistor is 14.3 cm^2^/Vs, and measured change on drain current (ΔI_ds_) at the gate voltage of 20 V is 1.2 μA under 780 nm LCP excitation. Therefore, the increased charge ($\Delta Q$) can be calculated as follow:

$$\Delta Q= \frac{2\times\Delta I_{ds}\times L}{\mu_{n}\times V_{g} \times W}= \frac{2 \times\left( 1.2 \times{10}^{-6} \right) \times50}{14.3 \times20 \times100}=4.2 \times{10}^{-9} \left[ C \right]$$

The total number of increased electrons at the channel is

$$n_{channel electron}= \frac{\Delta Q}{q}= \frac{4.2 \times{10}^{-9}}{1.6 \times{10}^{-19}}=2.625\times{10}^{10}$$

Without gate voltage, the device operates as a photoconductor, and the drain current can be obtained using following equation:

$$\Delta I_{ds}= \frac{1}{2}q\mu\Delta nE$$

The total number of increased electron under 780 nm LCP excitation is :

$$\Delta n= \frac{2\Delta I_{ds}}{q\mu E}= \frac{2 \times(7.88 \times{10}^{-10})}{(1.6 \times{10}^{-19}) \times14.3 \times\frac{10}{0.005}}=3.442\times{10}^{5}$$

The number of hot electrons that a single chiral gold nanoparticle contributed can be calculated by dividing previously calculated total number of electrons into the total number of chiral gold nanoparticles in active area. As the active area is 50 μm $\times$ 100 μm, and chiral gold nanoparticle is separated in 400 nm distance, the number of chiral gold nanoparticle in active area is calculated as 31250. Thus, the number of electrons contributed from each chiral gold nanoparticle under 780 nm LCP excitation is 2.625 $\times$ 10^10^ / 31250 = 8.4$\times$ 10^5^ at 20V gate voltage, and is 3.442 $\times$ 10^5^ / 31250 = 11 without gate voltage. Contribution of single chiral gold nanoparticle is presented below depending on wavelength, circular polarization state and gate voltage.

**Figure S5 |** The number of hot electrons that a single chiral gold nanoparticle contributed depending on wavelength and circular polarization state.

Figure S5 shows that the larger number of hot electrons are observed in 780 nm excitation rather than 635 nm excitation. Under 635 nm excitation, RCP excitation gives rise to larger number of hot electrons contribution than LCP excitation does, while the opposite trend is observed under 780 nm excitation. This trend is consistent with the photoresponsivity shown in Figure 2 and circular dichroism data displayed in Figure 1. In detail, tens of hot electrons contributed to photocurrent without gate voltage, while about hundred thousands of hot electrons contributed to photocurrent with gate voltage.

We further calculated the number of hot electrons compared to absorbed photons in chiral gold nanoparticle array.

The total number of photons absorbed by chiral gold nanoparticle array can be calculated as below.

$$\boldsymbol{n}_{\boldsymbol{photon}}\boldsymbol{=}\frac{\alpha\cdot\emptyset_{in}\cdot A}{qhv}$$

Where $\alpha$,$\emptyset_{in}$, $A$,$q$, and $hv$ are absorption, power density of incident light, active area of the device, charge of electron and energy of photon, respectively. We try to calculate the total number of photons at both wavelengths (635 nm (1.95 eV) and 780 nm (1.59 eV)). From the measured absorbance spectrum of chiral gold nanoparticle array, absorption ($\alpha$) was calculated about 74 % and 93 % at 635nm and 780nm wavelength, respectively. The active area ($A$) of device is 50 μm $\times$ 100 μm and laser power density ($\emptyset_{in}$) was 3.7 mW/cm^2^. Therefore, the number of absorbed photons in chiral gold nanoparticles array is

$$\boldsymbol{n}_{\boldsymbol{photon}\boldsymbol{at} \boldsymbol{780} \boldsymbol{nm}}=\frac{0.93\times\left( 3.7\times{10}^{-3} \right)\times(5 \times{10}^{-5})}{(1.6\times{10}^{-19})\times1.59} =6.8 \times{10}^{11}/s$$

$$\boldsymbol{n}_{\boldsymbol{photon}\boldsymbol{at} \boldsymbol{635} \boldsymbol{nm}}=\frac{0.74\times\left( 3.7\times{10}^{-3} \right)\times(5 \times{10}^{-5})}{(1.6\times{10}^{-19})\times1.95} =4.4 \times{10}^{11}/s$$

Hot electron contribution efficiency can be calculated as follows.

***Hot electron contribution efficiency (%)*** = $\frac{n_{channel electron}}{n_{photon}}$ $\times100$

In detail, about 10^-5^ % efficiency is calculated without gate voltage, while about 4% efficiency is calculated with gate voltage.


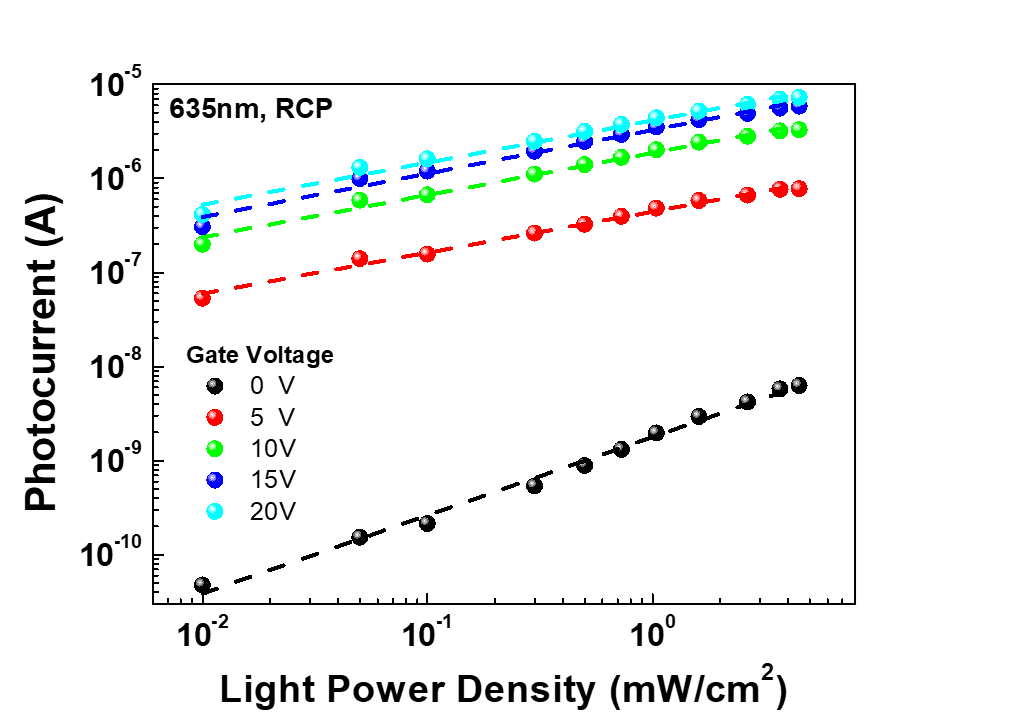


**Figure S6 |** Photocurrent measured under 635 nm RCP light illumination as a function of light power density**.**


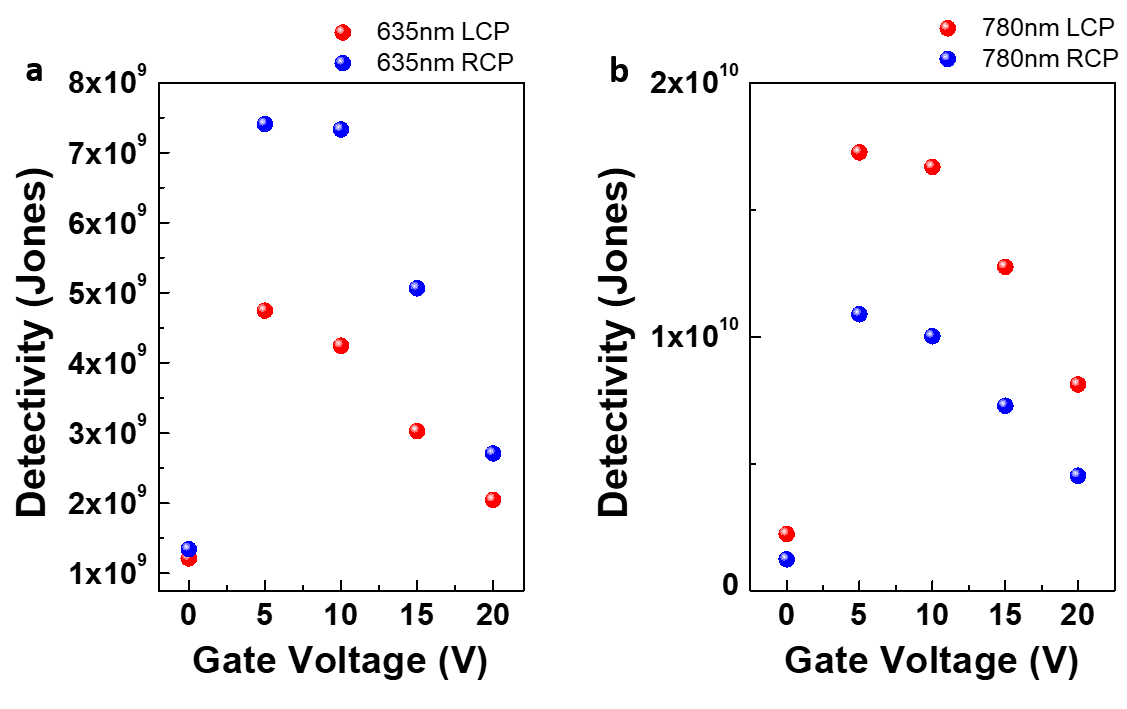


**Figure S7 | a, b,** Detectivity of the CPL detector depending on circular polarization states under 635 nm and 780 nm light illumination, respectively


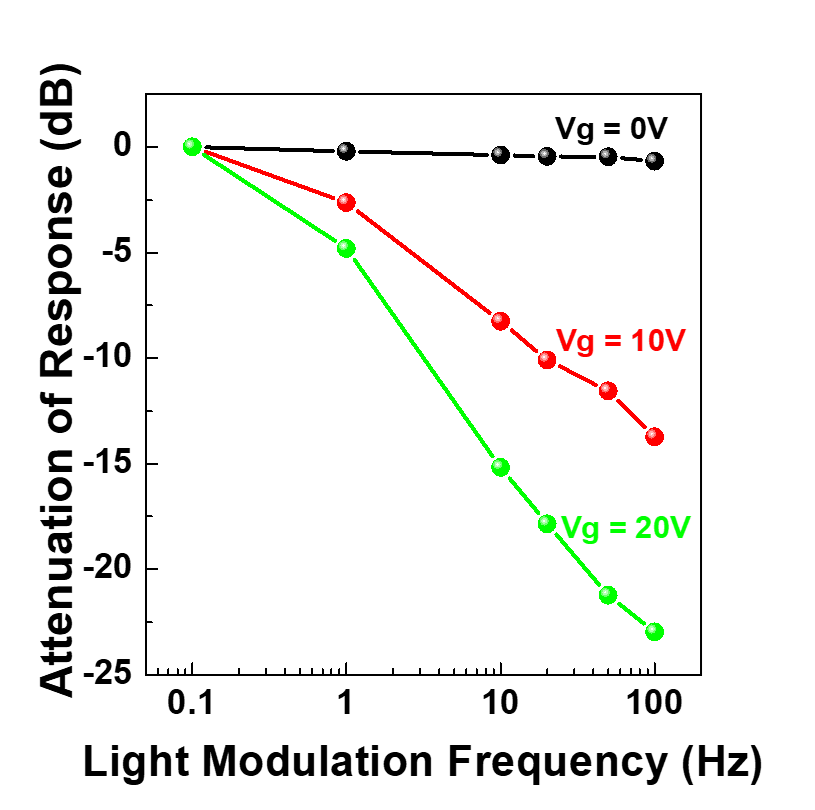


**Figure S8 |** Attenuation of the response with respect to the light modulation frequency of 635 nm RCP light.


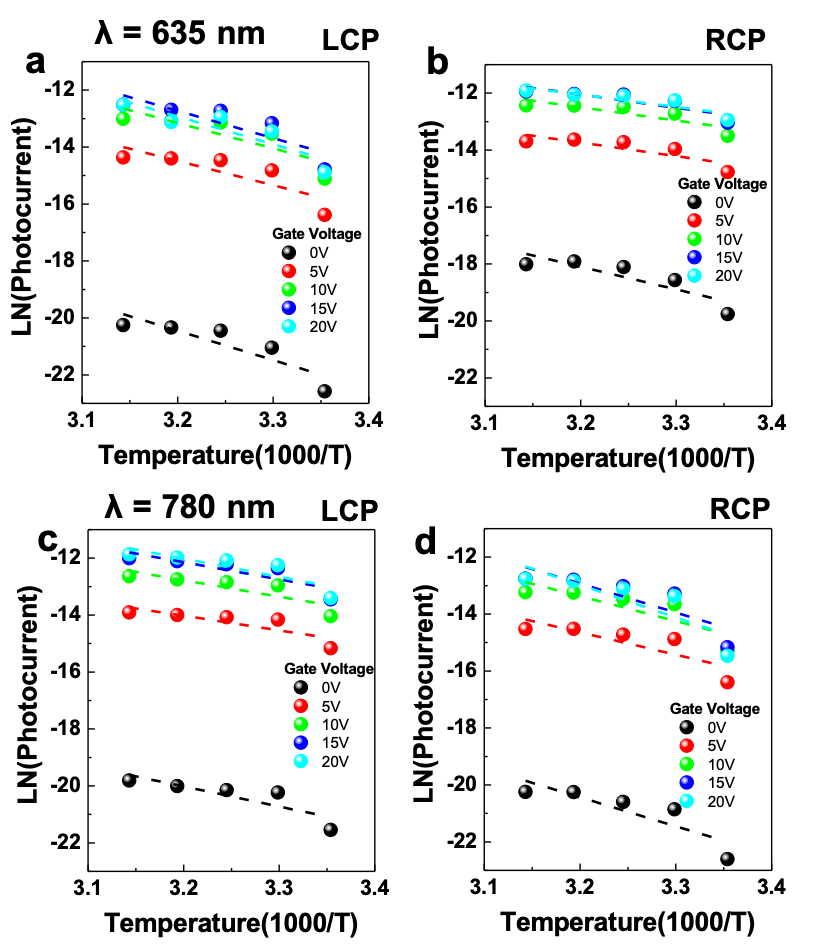


**Figure S9 | a, b,** Arrhenius plot of the CPL detector under 635 nm LCP and RCP laser excitation, respectively. **c, d,** Arrhenius plot of the CPL detector under 780 nm LCP and RCP laser excitation respectively.

**
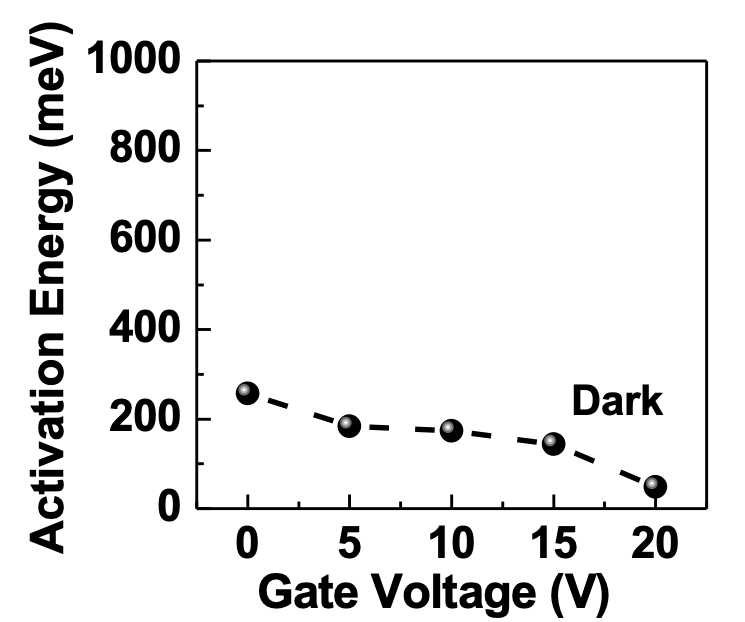
**

**Figure S10 |** Activation energy of the CPL detector under dark conditions with respect to the gate voltage.

**
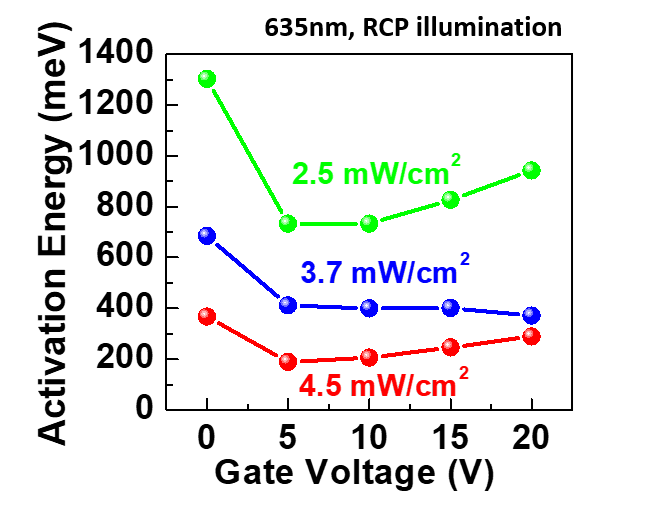
**

**Figure S11 |** Activation energy of the CPL detector under 635 nm RCP light illumination with different light power densities.


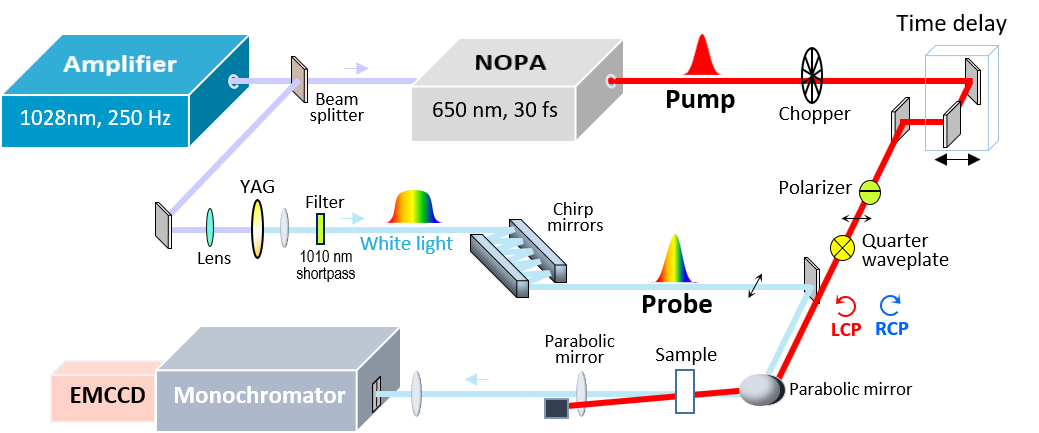


**Figure S12 |** Schematic of the broadband transient absorption measurement system.

**
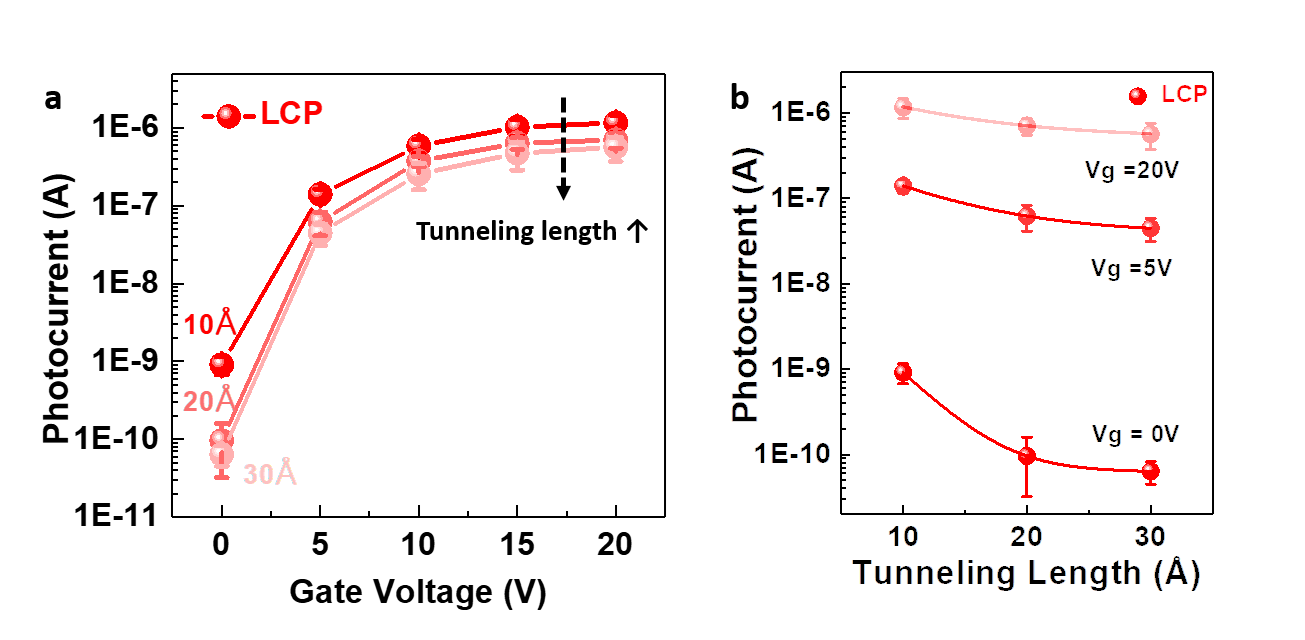
**

**Figure S13 | a,** Logarithmic photocurrent of the CPL-detecting transistor under 635 nm LCP light illumination when the device has a tunneling HfO_2_ film of various thickness at the interface between the chiral gold nanoparticles and InGaZnO layer. **b,** Corresponding photocurrent with respect to tunneling length, and the graph is fitted to exponential decay.


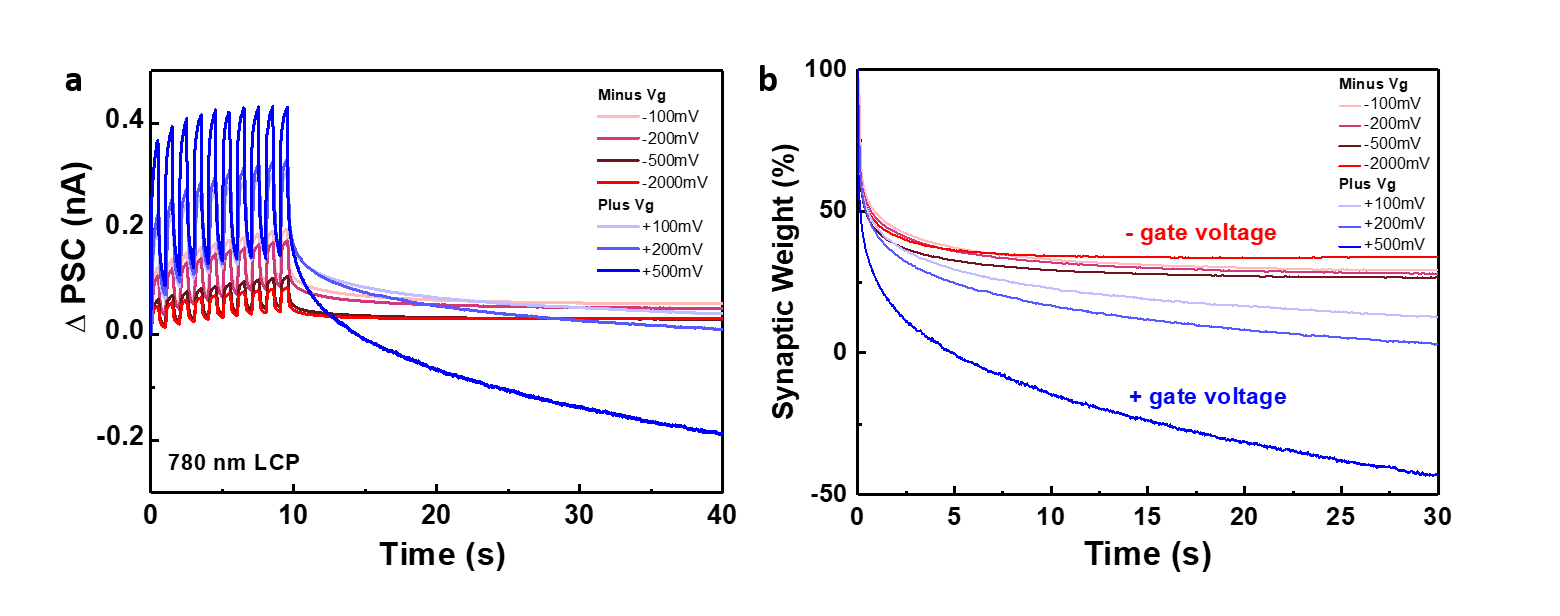


**Figure S14 | a,** Potentiation curve under 1 Hz LCP pulse at 780 nm wavelength and relaxation curve without light illumination when different gate voltages are biased. **b,** Magnified relaxation curve after ten light pulses are illuminated, in which synaptic weight is calculated by letting the current measured at the last light pulse be 100 %.

**
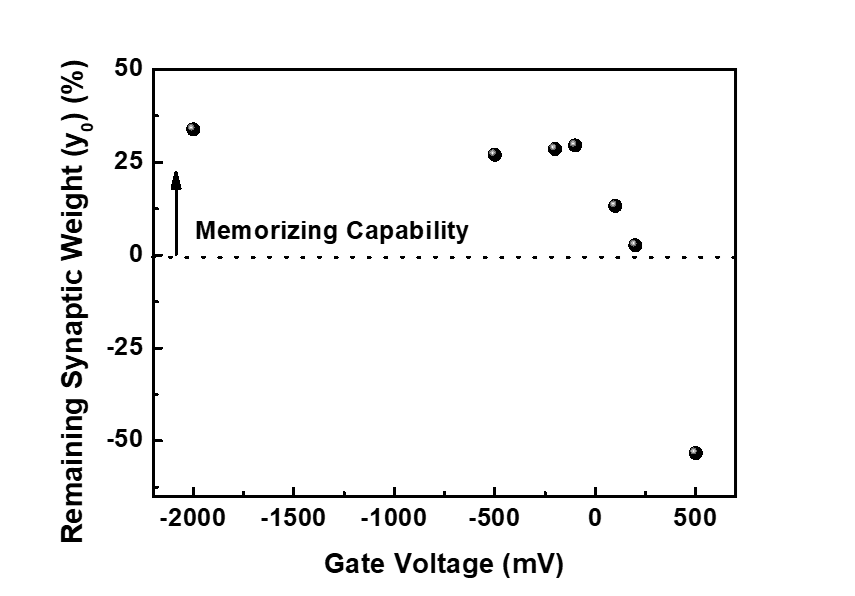
**

**Figure S15 |** Remaining synaptic weight as a function of gate voltage, in which minus gate voltage gives rise to higher remaining synaptic weight.


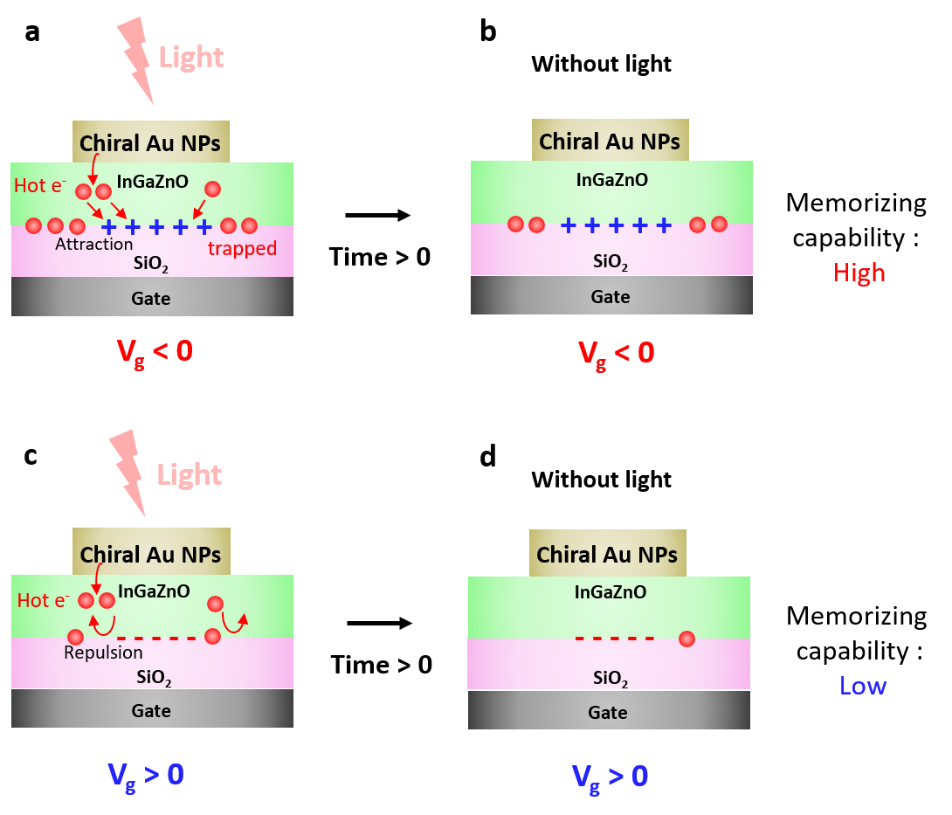


**Figure S16** | **a-b,** Schematic diagram of hot electron movement mechanism at the interface between SiO_2_ and InGaZnO when gate voltage is negatively biased under light excitation (a), and dark condition (b). **c-d,** Schematic diagram of hot electron movement when gate voltage is negatively biased under light excitation (c) and dark condition (d)
